# Supplementary material for: Prevalence of Chronic Heart Failure, Associated Factors, and Therapeutic Management in Primary Care Patients in Spain, IBERICAN Study
Source: J Clin Med. 2021 Sep 7;10(18):4036. doi: 10.3390/jcm10184036 (PMC8471153; doi:10.3390/jcm10184036)
Supplement: Supplementary file 1 [file jcm-10-04036-s001.zip › jcm-1334520-supplementary.pdf]

Characteristics of subjects with chronic heart failure

Table S1 describes the characteristics of patients according to the presence or absence of CHF. Patients with CHF were older and had more CVRF (AH, DM, dyslipidemia, obesity, and abdominal obesity) (p<0.001), more TOD (left ventricular hypertrophy, pulse pressure, and pathological ankle-brachial index) (p<0.001), and more associated CVD (IHD, stroke, atrial fibrillation, and peripheral vascular disease) (p<0.001). Likewise, they received more drug treatments with antihypertensives, lipid lowering drugs, antiplatelets, and anticoagulants (p<0.001).

Table S1. Clinical characteristics of patients with and without chronic heart failure*

**Variables**	**No CHF (n=7.817)**	**CHF (n=249)**	p
Median age in years (IQR)	*59.0 (48.0, 69.0)*	*72.0 (64.0, 80.0)*	<0.001
Men<br>Women	3550 (45.4%)<br>4267 (54.6%)	117(47.0%)<br>132 (53.0%)	0.623
Median body mass index (Kg/m^2) (IQR)	*27.8 (24.9, 31.2)*	*29.8 (26.6, 33.9)*	<0.001
Median waist circumference (IQR)	*96.0 (87.0, 105.0)*	*100.0 (92.0, 110.0)*	<0.001
Median clinical SBP (mmHg) (IQR)	*130.0 (120.0, 140.0)*	*134.0 (120, 145.0)*	0.530
Median clinical DBP (mmHg) (IQR)	*77.0 (70.0, 83.0)*	*77.0 (69.0, 85.0)*	<0.001
**Analytical parameters**			
Median glucose (mg/dl) (IQR)	*95.0 (86.0, 108.0)*	*101.0 (89.0, 124.0)*	<0.001
Median total cholesterol (mg/dl (IQR)	*194.0 (169.0, 220.0)*	*180.5 (157.0, 212.0)*	<0.001

Median LDL-cholesterol (mg/dl) (IQR)	*115.0 (93.0, 139.0)*	*106.0 (83.0, 134.0)*	<0.001
Median HDL-cholesterol (mg/dl) (IQR)	*53.0 (44.0, 64.0)*	*48.0 (40.0, 57.0)*	<0.001
Median triglycerides (mg/dl) (IQR)	*107.0 (78.0, 150.0)*	*124.0 (92.4, 188.5)*	<0.001
Median HbA1c (%) (SD); median (IQR)	*6.8 (6.2, 7.6)*	*7.2 (6.3, 8.0)*	0.03
Median Creatinine (mg/dl) (IQR)	*0.8 (0.7, 0.9)*	*0.9 (0.8, 1.2)*	<0.001
Median uric acid (mg/dl) (IQR)	*5.1 (4.2, 6.1)*	*6.0 (4.7, 7.1)*	<0.001
Median estimated glomerular filtration rate (CKD-EPI) (IQR)	*90.6 (77.6, 101.4)*	*68.5 (55.3, 86.7)*	<0.001
Mean albumin-to-creatinine ratio (SD)	14.5 (62.4)	29.1 (107.5)	<0.001
**Cardiovascular risk factors**			
Family history of early CVD	1093 (15.3%)	73 (30.7%)	<0.001
Hypertension	3660 (45.4%)	200 (80.3%)	<0.001
Diabetes mellitus	1519 (17.9%)	104(41.8%)	<0.001
Smoking	1386 (18.4%)	24 (29.6%)	<0.001
Dyslipidemia	3867 (49.6%)	177(71.1%)	<0.001
General obesity	2690 (34.7%)	123 (49.4%)	<0.001
Abdominal obesity	4256 (55.2%)	170 (68.5%)	<0.001
Sedentary lifestyle	2227 (28.7%)	124 (49.8%)	0.023
Metabolic syndrome	3051 (39.0%)	161 (64.7%)	<0.001
**Target organ damage**			
Left ventricular hypertrophy	241 (3.1%)	76 (30.5%)	<0.001
Ankle-brachial index < 0.9	126 (1.6%)	9 (3.6%)	0.015
Pulse pressure (> 65 years) > 60	1258(16.1%)	93 (37.3%)	<0.001

Microalbuminuria (30-300)	521 (6.7%)	37 (14.9%)	<0.001
Glomerular filtration rate CKD-EPI (30-60 ml/min)	507 (6.6%)	70 (28.6%)	<0.001
**Associated clinical disease**			
Coronary disease	523 (6.7%)	61 (24.5%)	<0.001
Stroke	291 (3.7%)	32 (12.9%)	<0.001
Peripheral artery disease	226 (2.9%)	39 (15.7%)	<0.001
Atrial fibrillation	243 (3.1%)	95 (38.2%)	<0.001
Albuminuria (> 300 mg/g)	49 (0.6%)	8 (3.2%)	<0.001
GFR < 30 ml/min	76 (1.0%)	11 (4.5%)	<0.001
Advanced retinopathy	36 (0.5%)	11 (4.4%)	<0.001
**Treatments**			
Thiazide diuretics	1129 (30.7%)	42 (27.0%)	0.004
Loop diuretics	157 (4.3%	69 (34.1%)	<0.001
ACEI	1399 (38.1%)	87 (42.8%)	<0.001
ARB	1557 (42.4%)	84 (41.3%)	0.811
Beta-blockers	629 (17.1%)	108 (54.7%)	<0.001
MRA	79 (2.1%)	32 (16.8%)	<0.001
Calcium antagonists	828 (22.5%)	53 (26.7%)	0.191
Alpha-blockers	109 (3.0%)	11 (5.4%)	0.044
Statins	2657 (68.4%)	147 (82.8%)	<0.001
Antiplatelets	1040 (13.3%)	87 (34.9%)	<0.001
Anticoagulants	311 (4.0%)	109 (43.8%)	<0.001
Antihypertensives (Mean)	1.6 (0.9%)	2.4 (0.9%)	<0.001

Antidiabetics (Mean)	1.5 (0.9%)	1.7 (0.9%)	0.108

* The data are expressed as total number of individuals who presented definition criteria of the variable and percentage for qualitative variables, and as mean (standard deviation) and median (interquartile range) for quantitative variables. CHF: chronic heart failure; SD: standard deviation; SBP: systolic blood pressure; DBP: diastolic blood pressure; HbA1c: glycosylated hemoglobin; PVD: peripheral vascular disease; CVD: cardiovascular disease; MRA: mineralocorticoid receptor antagonist; ACEI: angiotensin-converting enzyme inhibitors; ARB: angiotensin II receptor blockers.

Table S2 shows clinical characteristics according to EF of the patients included. Patients with CHF with rEF mainly had IHD and peripheral vascular disease (PVD) in their history. In patients with pEF, AH was the most important pre-existing condition, they presented higher levels of SBP and DBP, and more TOD.

Table S2. Clinical characteristics of patients with chronic heart failure according to ejection fraction*

**Variables**	**Total** N=249	**CHF with reduced LVEF** n= 95	**CHF with preserved LVEF** n= 154	**p**
Median Age in years (IQR)	*72.0 (64.0, 80.0)*	71.0 (60.0, 80.0)	73.0 (66.0, 80.0)	0.181
Sex<br>-Man<br>-Woman	 117 (47.0%)<br>132 (53.0%)	 50 (52.6%)<br>45 (47.4%)	 67 (43.5%)<br>87 (56.5%)	 0.161
Median BMI (kg/m^2) (IQR)	*29.8 (26.6, 33.9)*	*30.3 (27.3, 33.5)*	*26.7 (26.2, 34.0)*	0.689

Median Waist circumference (IQR)	*100.0 (92.0, 110.0)*	*102.0 (94.0, 111.0)*	*99.0 (90.0, 110.0)*	0.096
Median clinical SBP (mmHg) (IQR)	*134.0 (120.0, 145.0)*	*130.0 (120.0, 143.0)*	*135.0 (125.0, 145.0)*	0.169
Median clinical DBP (mmHg) (IQR)	*77.0 (69.0, 85.0)*	*73.0 (65.0, 82.0)*	*78.0 (70.0, 86.0)*	0.055
**Analytical parameters**				
Median glucose (mg/dl) (IQR)	*101.0 (89.0, 124.0)*	*104.0 (88.0, 126.0)*	*100.0 (89.0, 119.0)*	0.125
Median HbA_1c (%) in diabetes (IQR)	*7.2 (6.3-8.0)*	*7.2 (6.3-8.1)*	*7.3 (6.3-7.9)*	0.822
Median total cholesterol (mg/dl) (IQR)	*180.5 (157.0, 212.0)*	*176.5 (157.0, 212.0)*	*182.5 (157.0, 212.0)*	0.687
Median LDL-cholesterol (mg/dl) (IQR)	*106.0 (83.0, 134.0)*	*104.0 (79.0, 136.0)*	*106.0 (85.0, 134.0)*	0.826
Median HDL-cholesterol (mg/dl) (IQR)	*48.0 (40.0, 57.0)*	*51.0 (42.0, 61.0)*	*45.0 (40.0, 57.0)*	0.089
Median triglycerides (mg/dl) (IQR)	*124.0 (92.4, 188.5)*	*120.5 (86.0, 183.0)*	*130.5 (97.0, 193.0)*	0.089

Median creatinine (mg/dl) (IQR)	*0.96 (0.80, 1.17)*	*0.96 (0.81, 1.12)*	*0.90 (0.80, 1.20)*	0.865
Median estimated glomerular filtration rate (CKD-EPI) (IQR)	*68.5 (55.3, 86.7)*	*69.4 (56.3, 87.3)*	*68.2 (53.8, 85.9)*	0.522
Albumin-to-creatinine ratio	29.1 (107.5)	31.5 (74.0)	27.7 (123.9)	0.790
**CVRF**				
Arterial hypertension	200 (80.3%)	75 (78.9%)	125 (81.2%)	0.668
Diabetes mellitus	104 (41.8%)	41.0 (43.2%)	63 (40.9%)	0.727
Smoking	24 (9.6%)	11 (11.6%)	13 (8.4%)	0.415
Dyslipidemia	177 (71.1%)	68 (71.6%)	109 (70.8%)	0.892
General obesity	123 (49.4%)	49 (51.6%)	74 (46.1%)	0.589
Abdominal obesity	170 (68.5%)	64 (67.4%)	106 (69.3%)	0.752
Sedentary lifestyle	124 (49.8%)	55 (57.9%)	69 (4.8%)	0.045
**TOD**				
LVH	76 (30.5%)	27 (28.4%)	49 (31.8%)	0.572
Microalbuminuria	37 (14.9%)	15 (15.8%)	22 (14.3%)	0.728
Ankle-brachial index < 0.9	9 (3.6%)	2 (2.1%)	7 (4.5%)	0.316
PP (> 65 years) > 60	93 (37.3%)	31 (32.6%)	62 (40.3%)	0.227
**Associated clinical disease**				
Ischemic heart disease	61 (24.5%)	32 (33.7%)	29 (18.8%)	0.008

Stroke	32 12.9%)	13 (13.7%)	19 (12.3%)	0.758
PVD	39 (15.7%)	21 (22.1%)	18 (11.7%)	0.028
Atrial fibrillation	95 (38.2%)	39 (41.1%)	56 (36.4%)	0.458
Albuminuria (> 300 mg/g)	8 (3.2%)	4 (4.2%)	4 (2.6%)	0.046
GFR < 30 ml/min	11 (4.5%)	7 (7.5%)	4 (2.6%)	0.048
**Drug treatments**				
Loop diuretics	69 (34.1%)	26 (33.8%)	43 (34.4%)	0.926
Thiazide diuretics	42 (27.0%)	18 (23.4%)	24 (30.6%)	0.477
MRA	32 (16.8%)	16 (20.8%)	16 (12.8%)	0.131
Beta-blockers	108 (54.7%)	46 (59.7%)	62 (49.6%)	0.160
ACEI	87 (42.8%)	32 (41.6%)	55 (44.0%)	0.733
ARB	84 (41.3%)	31 (40.3%)	53 (42.4%)	0.764
Calcium antagonists	53 (26.7%)	22 (28.6%)	31 (24.8%)	0.554
Alpha-blockers	11 (5.4%)	4 (5.2%)	7 (5.6%)	0.901
Statins	147 (82.8%)	60 (85.7%)	87 (79.8%)	0.314
Metformin	66 (62.7%)	26 (61.9%)	40 (63.5%)	0.869
Sulfonylureas	13 (11.9%)	4 (9.5%)	9 (14.3%)	0.467

* The data are expressed as total number of individuals who presented definition criteria of the variable and percentage for qualitative variables, and as mean (standard deviation) and median (interquartile range) for quantitative variables. CHF: chronic heart failure; LVEF: left ventricular ejection fraction; SBP: systolic blood pressure; DBP: diastolic blood pressure; BMI: body mass index; HbA1c: glycosylated hemoglobin; CVRF: cardiovascular risk factors; TOD: target organ damage; GFR: estimated glomerular filtration rate by CKD-EPI; PVD: peripheral vascular disease; LVH: left ventricular hypertrophy; PP: pulse pressure; MRA: mineralocorticoid receptor antagonist; ACEI: angiotensin-converting enzyme inhibitors; ARB: angiotensin II receptor blockers.
